# Behavioural Change Techniques in Health Coaching-Based Interventions for Type 2 Diabetes: A Systematic Review and Meta-Analysis

**DOI:** 10.1186/s12889-022-14874-3

**Published:** 2023-01-13

**Authors:** Abdullah N. Almulhim, Hannah Hartley, Paul Norman, Samantha J. Caton, Onur Cem Doğru, Elizabeth Goyder

**Affiliations:** 1 School of Health and Related Research, The University of Sheffield, 30 Regent St, Sheffield, S1 4DA UK; 2 Public Health Department, College of Health Sciences, Saudi Electronic University, Riyadh, 13316 Saudi Arabia; 3 Yorkshire Quality and Safety Research Group, Bradford Institute for Health Research, Temple Bank House, Bradford Royal Infirmary, Bradford, BD9 6RJ UK; 4 Department of Psychology, The University of Sheffield, Cathedral Court, The University of Sheffield, Vicar Ln, Sheffield, S1 2LT UK; 5 Department of Psychology, Afyon Kocatepe University, Gazlıgöl St, 03200 Afyonkarahisar, Turkey

**Keywords:** Health behaviour change, Health coaching, Self-management, Behaviour change techniques, Type 2 diabetes

## Abstract

**Background:**

Given the high rates globally of Type 2 Diabetes Mellitus (T2DM), there is a clear need to target health behaviours through person-centred interventions. Health coaching is one strategy that has been widely recognised as a tool to foster positive behaviour change. However, it has been used inconsistently and has produced mixed results. This systematic review sought to explore the use of behaviour change techniques (BCTs) in health coaching interventions and identify which BCTs are linked with increased effectiveness in relation to HbA1C reductions.

**Methods:**

In line with the PICO framework, the review focused on people with T2DM, who received health coaching and were compared with a usual care or active control group on HbA1c levels. Studies were systematically identified through different databases including Medline, Web of science, and PsycINFO searches for relevant randomised controlled trials (RCTs) in papers published between January 1950 and April 2022. The Cochrane collaboration tool was used to evaluate the quality of the studies. Included papers were screened on the reported use of BCTs based on the BCT taxonomy. The effect sizes obtained in included interventions were assessed by using Cohen’s d and meta-analysis was used to estimate sample-weighted average effect sizes (Hedges’ g).

**Results:**

Twenty RCTs with a total sample size of 3222 were identified. Random effects meta-analysis estimated a small-sized statistically significant effect of health coaching interventions on HbA1c reduction (*g*_+_ = 0.29, 95% CI: 0.18 to 0.40). A clinically significant HbA1c decrease of ≥5 mmol/mol was seen in eight studies. Twenty-three unique BCTs were identified in the reported interventions, with a mean of 4.5 (SD = 2.4) BCTs used in each study. Of these, *Goal setting (behaviour) and Problem solving* were the most frequently identified BCTs. The number of BCTs used was not related to intervention effectiveness. In addition, there was little evidence to link the use of specific BCTs to larger reductions in HbA1c across the studies included in the review; instead, the use of *Credible source* and *Social reward* in interventions were associated with smaller reductions in HbA1c.

**Conclusion:**

A relatively small number of BCTs have been used in RCTs of health coaching interventions for T2DM. Inadequate, imprecise descriptions of interventions and the lack of theory were the main limitations of the studies included in this review. Moreover, other possible BCTs directly related to the theoretical underpinnings of health coaching were absent. It is recommended that key BCTs are identified at an early stage of intervention development, although further research is needed to examine the most effective BCTs to use in health coaching interventions.

**Trial registration:**

https://www.crd.york.ac.uk/prospero/display_record.php?ID=CRD42021228567.

**Supplementary Information:**

The online version contains supplementary material available at 10.1186/s12889-022-14874-3.

## Background

Type 2 Diabetes Mellitus (T2DM) is a chronic condition that is a significant public health concern. It was estimated that 462 million of the global population had T2DM in 2017, with this figure projected to increase by 6.28% up to 491 million people globally by 2030 [1]. T2DM is associated with an increased risk of co-morbidity and other health implications, such as heart and stroke disorders, eye problems and complications with hearing, kidney failure, nerve injury, amputations, oral issues, and foot problems [2]. Having a raised body mass index (BMI), low physical activity levels and unhealthy dietary patterns are key contributing factors of developing T2DM [3]. Fortunately, these lifestyle behaviours are modifiable through intervention which can reduce the risk of developing the condition [4]. However, recent economic growth has generated an obesogenic environment, resulting in the widespread availability of affordable unhealthy foods and an increase in sedentary lifestyles. This perpetuates unhealthy dietary patterns and low physical activity levels, and presents challenges to attempts to modify lifestyle behaviours to reduce the risk of developing T2DM [5].

Supporting people with T2DM to self-manage their condition is considered key to successfully changing lifestyle behaviours to reduce the risk of T2DM associated health implications [3]. Successful self-management and behaviour change in people with T2DM can significantly reduce or delay chronic conditions associated with T2DM by at least 75% [6]. This has led many healthcare systems to adapt their care of T2DM to focus on self-management and individualised behaviour change, requiring a more client-centred approach [7]. Individualised, self-management approaches for non-communicable conditions such as T2DM are increasingly being advocated [8, 9]. Among those at high risk, randomized controlled trials have shown that altering one’s lifestyle can reduce the risk of acquiring diabetes by 58% in people with impaired glucose tolerance [10, 11]. To date, self-management behaviour change T2DM interventions can be characterized mainly by their emphasis on the role of education and motivation as strategies for behaviour change. These interventions have resulted in only short term behaviour change, with poor effects in enabling targeted people to maintain the self-management skills needed to make long-term behaviour change [12–15].

Health coaching based interventions have been proposed as a more appropriate approach in achieving long term behaviour change for the self-management of T2DM [16]. Health coaching is a one-to-one support intervention style described by Wolever et al. as “a patient-centred approach wherein patients at least partially determine their goals, use self-discovery or active learning processes together with content education to work toward their goals, and self-monitor behaviours to increase accountability, all within the context of an interpersonal relationship with a coach” [17]. Health coaching grew out of counselling and health education fields [18], and has been widely used in different contexts as an intervention for addressing lifestyle-related conditions, including T2DM [16]. The growing acceptability of health coaching aligns with the shift towards a more person focussed self-management model in healthcare settings [6].

Many studies have shown the efficacy of using health coaching with different chronic conditions, including T2DM [19]. However, recent systemic reviews of randomised controlled trials utilising health coaching have reported mixed results, with some reporting that health coaching is effective, while others claim it is ineffective [19, 20]. One of the contributing factors of inconsistent findings across these studies is the lack of consensus on the active ingredients and content to be included in health coaching interventions [17]. In general, a lack of guidance, inappropriately selected intervention components and variation in the reporting of outcomes has been suggested to contribute to the mixed evidence for effectiveness of health coaching interventions [21–23]. Consequently, there is currently no consensus in the literature on designing an effective health coaching intervention, including the selection of a suitable theoretical basis and active components for behavior change [20]. In the absence of such consensus, there is uncertainty towards which coaching methods are more appropriate and effective to replicate and use; this includes the intervention content, duration, length, and mode of delivery of sessions [24].

To support the systematic application of active components to change behaviours, the behaviour change technique taxonomy (BCTTv1), can be applied. The BCTTv1 is an extensive taxonomy of behaviour change techniques (BCTs) that can be utilised as active behaviour change components in behaviour change interventions [25]. A BCT is defined as “an observable, replicable, and irreducible component of an intervention designed to alter or redirect causal processes that regulate behaviour” [25]. The taxonomy consists of 93 BCTs clustered into16 groups. BCTs can be used with numerous theoretical perspectives, in isolation or in combination with other BCTs. The development and evaluation of interventions incorporating BCTs may enable researchers to systematically apply, identify and report the key ‘active ingredients’ in interventions [25]. This, in turn, may generate understanding of effective active components in behavior change interventions targeting T2DM and increase the possibility of replication [25].

A number of reviews have highlighted that the use of BCTs in interventions that target behaviours related to physical activity and maintaining a healthy weight may result in better management of HbA1c in people with T2DM [26]. For example, employing certain BCTs in dietary interventions, such as *instruction on how to perform a behavior*, *demonstration of the behavior*, *behavioral practice/rehearsal*, and *action planning*, has linked to a greater impact on HbA1c levels for people with T2DM [27]. Similarly, the use of two BCTs, *goal setting* and *review of behavior/outcome goals,* has been shown to have a positive impact on reducing fat intake for people with T2DM [28]. Another review of web-based interventions found that using the BCTs of *feedback on behavior, information about health consequences*, *problem solving*, and *self-monitoring of behavior*, was linked to improvements in changing behavior, psychological conditions clinical parameters in people with T2DM [29]. The BCT of *social support, natural consequences, antecedents, associations, shaping knowledge, social support* and *goals* were used most frequently in interventions that target T2DM [30]. A recent review urged employing the following BCTs when developing psychological interventions that target T2DM to improve HbA1c; *social support (unspecified), problem solving*, and *goal setting (behavior)* [31]. The findings of these reviews indicate that a detailed analysis of the BCTs used in health coaching interventions for T2DM, and the extent to which they are associated with greater reductions in HbA1c, is likely to aid the development and replication of effective health coaching interventions for T2DM.

This review therefore aimed to bridge the current gaps in knowledge by addressing the four main objectives. It sought to: 1) Assess health coaching intervention content in relation to reporting sufficient and precise descriptions of used behaviour change theories and BCTs; 2) Identify the BCTs used in health coaching interventions; 3) Assess whether the inclusion of specific BCTs are associated with larger effect sizes of interventions; and 4) Explore key intervention characteristics and methodological characteristics and their association with reported effects, including coaching intervention duration, length of sessions, mode of delivery, and demographic variables.

## Method

This systematic review and meta-analysis was reported following the Preferred Reporting Items for Systematic reviews and Meta-Analyses (PRISMA) statement [32]. The review protocol was registered in the International Prospective Register of Systematic Reviews (PROSPERO) database (CRD42021228567).

### Search strategy and inclusion/exclusion criteria

To identify the relevant literature, a series of systematic searches was conducted on PsycINFO, Medline (Ovid) and the Web of Science. The searches were conducted using the keywords and their combinations. Medline key search terms included: “type II diabetes mellitus,” “non-insulin dependent diabetes mellitus,” “Diabetes Mellitus, Type 2/ or diabetes,” “Coaching,” “Health Coaching,” and “personal coach*” (see Supplementary Material 1 for more details on Medline search strategy). A manual back chaining was utilised as an additional step to supplement the database searches find relevant literature. This involved examining the list of all the references in the included studies, including potential citations within each article and other relevant reviews.

The current review focused on people with T2DM (Population), who received health coaching (Intervention) and who were compared with a usual care or active control group (Comparison) on HbA1c levels (Outcome). Studies were only included if they were peer-reviewed RCTs, reported changes in HbA1c, published in English from January 1950 and April 2022, included participants aged 18 years or older and employed health coaching to influence T2DM. For the purpose of this review, health coaching was defined as using client-centred sessions in which the coach uses coaching skills and techniques to enable the client to engage and work toward their intended goals. The start date of searching was purposely selected to cover all coaching terms, such as health counselling, coaching, personal coaching, and health promotion in published studies from the emerging time of health coaching in the early 1950s. Articles were excluded if participants did not have a diagnosis of T2DM; were not subject to health coaching interventions; self-management was not the targeted behaviour; included other variations of diabetes, e.g., gestational diabetes or type 1 diabetes mellitus; and HbA1c was not reported as an outcome measure. This review therefore included interventions that investigated the effectiveness of using the health coaching approach as a tool to impact the self-management of T2DM. Only RCT studies were included to explore effectiveness of the interventions and minimize the risk of bias [33].

### Study selection and data extraction

Search results were initially screened against the inclusion criteria at title and abstract level. Full texts of these articles were screened next. Screening was completed independently by two researchers (AA, HH). The first author extracted data from the included studies, and then the second author reviewed the data for verification. Conflicts resolved by discussion between two reviewers (AA, HH). An independent reviewer (PN) conducted an additional step to double-check the extracted data. Data were systematically extracted using a prespecified extraction form (see Table 1 and Supplementary Material 2). Related studies (e.g., published protocols) were reviewed to extract further information. Relevant study information from the included articles was reviewed and data extracted (e.g., design, the theory or model used, BCTs, intervention structure, target behaviours, and outcome parameters) by two reviewers (AA, HH). The RCTs included were coded as to theories and BCTs used in the interventions as well as reported effects on glycaemic control. RCTs were also coded according to the modes of delivery, length and duration of the health coaching sessions.

**Table 1:** Description of included studies in the systematic review

Study, Country, and Objectives	Sample (completed), Mean Age; Female %	Study Duration (M) (Intervention+ Follow-ups)	Delivery mode	Intervention providers	Measurements (n)	Results	Control group
Frosch et al. (2011), U S[34] To assess participants’ improvement in self-care behaviours, level of HbA1C, lipid and BP levels at 6 months	201 (201); 55.5; 48.5	6	TEL	Nurse educators	(3), A1c, lipid and blood pressure	Decline in HbA1c at 6 months in both groups (*P* < 001),	Educational brochure
Glasgow et al. (2006), U S[35] To assess the impact of a computer-assisted intervention on T2DM self-management	335 (299); 61.5; 50.2	2	FTF,TEL,ERPM/EA	Health educators	(7) FVSS, Daily fat intake, HbA1c, Cholesterol, PHQ, DDS, BMI	There was a decline in HbA1c favouring intervention group, but these differences did not reach significance	Computer-enhanced, Usual care
Kim et al., (2015), U S[36] To assess effectiveness of a community-based, culturally tailored, program in T2DM patients	250 (209); 58.7; 43	12	TEL, GER	Nurses and community health workers	(8) HbA1c, Triglyceride, Cholesterol, Blood pressure, diabetes-related quality of life, self-efficacy, adherence to diabetes management regimen, and health literacy	The difference between the two groups was statistically significant favouring the intervention group (reductions in HbA1c: 1 .0–1.3% compared to the control group with reductions of 0.5–0.7%)	Educational brochure
McKay et al. (2002), U S[37] To assess the impact of using an internet-based in improving diabetes self-management	160 (133); 59.3; 53.1	3	ERPM/EA	Health coach	(6) HbA1c, Fat intake, Poor dietary practices, Depression symptoms, Psychological well-being (SF-12), Total cholesterol	There was an improvement but not statistically significant difference favouring coaching group in relation to HbA1c	Information- only reading
Ruggiero et al. (2010), U S[38] To assess the effect of the intervention delivered by medical assistant coach on HbA1C compared with usual care group	50 (42); 65.8; 66	6	FTF,TEL	Medical assistants	HbA1c	HbA1C level decreased across the intervention group (MAC), but it was not significant between groups	Treatment as usual
Sacco et al. (2009), U S[39] To evaluate the effects of telephone-bases coaching provided by professionals on T2DM, including diabetes adherence and control, diabetes-related complications, and diabetes distress	62 (48); 52; 58	6	TEL	University students	(9) HbA1c, Diet, Exercise, Foot care, Depression, Self-efficacy, HTS, RSC, ASC	HbA1C decreased in the coaching group (M = 7.4%; SD = 1.12), but was not statistically significant	Usual Care
Thom et al. (2013), U S[40] To determine how clinic-based peer health coaching affects the management of uncontrolled T2DM in low-income populations	299 (236); 55.2; 52	6	FTF,TEL	Peers	(4) HbA1c, BMI, LDL, SBP	The difference was statistically significant between the two groups favouring the coaching group (HbA1C decreased by 1.07%) Whereas the reduction was 0.3% in the control group	Usual Care
Whittemore et al. (2004), U S[41] To assess the effect nurse-coaching intervention on T2DM	53 (49); 57.6; 100	6	FTF,TEL,ERPM/EA	Nurses	(5) HbA1C, BMI, Dietary, Exercise, Distress	A difference between the two groups was documented at 3 months in HbA1C levels favouring the coaching group, but the difference was not statistically significant	Usual Care
Willard-grace et al. (2015), U S[42] To assess impacts of health coaching in the control of T2DM, Hypertension, and Hyperlipidemia compared with usual care	144 (132); NA; NA	12	FTF, TEL	Medical assistants	(4) HbA1c, HDL, LDL, SBP	Intervention group was as twice as many patients in control arm achieved the HbA1c goal (48.6% vs 27.6%, *P* = .01). The difference was statistically significant	Usual Care
Wolever et al. (2010), U S[43] To evaluate the impact of integrative health coaching on various T2DM patient variables	56 (49); 53; 77	6	TEL	Psychologist and social worker	(10) HbA1c, ASK-20, MAS; PAM; ADS, BFS, ISEL-12, PSS-4, SF-12, Exercise	HbA1c was reduced in the intervention group significantly by 0.64% (from 8.9 1.78% at baseline to 8.3 1.76%; *P* = .030; Cohen d = .34).	Usual Care
Chen et al., (2016), Taiwan [44] To evaluate changes in HbA1c for group provided care by pharmacist compared usual care without a pharmacist	100 (100); 72.5;50	6	FTF,TEL	Certified diabetes educator Pharmacist	(1), Change in A1c level (6 months)	HbA1c level significantly decreased (0.83%) for the intervention group with an increase of 0.43% for the usual care arm (*P* ≤ 0.001).	Usual Care
Lin et al., (2021), Taiwa n[45] To explore the impact of health coaching on A1c and diet for patients with T2DM	114(114)45;49	6	FTF, TEL	Health Coach	(8) HbA1c, Daily calorie intake, Whole grains, Meats and protein, Milk and dairy products, Vegetables, Fruits, Fats and oils	Patients with type 2 diabetes who underwent a 6-month health coaching program saw a significant reduction in HbA1c by 0.62% (*P* < 0.01)	Usual care
Basak Cinar & Schou (2014), Turkey [46] To assess the difference in outcomes between health coaching group compared with usual health education for T2DM	186 (162^a^); NA; NA 100	16 M (10+ 6)	FTF, TEL	Dental professional	(3), HbA1C, CAL and TBSES	Significant differences found for HbA1C in Health coaching group, (*P* < 0.05)	Health education
Sherifali et al., (2021), Canad a[47] To assess the impact of telephone health coaching on A1c for patients with T2DM	365(365) 57;50	12 M (6 + 6)	TEL	Registered nurse/certified diabetes educator	(2) HbA1c, ADDQoL-19	HbA1c was reduced in the intervention group significantly by 1.78% (*P* < 0.005)	Usual diabetes education
Cho et al. (2011), Kore a[48] To assess impact of health coaching on HbA1c improvement after 3 months	71 (64); 64.2; NA	3	FTF, ERPM	Physicians and nurses	(2), HbA1c, cholesterol	HbA1c level was significantly decreased for intervention group (reduced from 8.0 to 7.5%) *P* < 0.0. In control group HbA1c reduced from 8.0 to 7.8%, *P* = 0.11)	Diabetes education
Holmen et al. (2014), Norway [49] To assess effectiveness of using phone-based self-management system used by a diabetes specialist on HbA1c, diabetes self-management, and improvement in quality of life	151 (120); 57.0; 41	12 M(4+ 8)	TEL,ERPM/EA	T2DM specialist nurse	(9) HbA1c, BMI, PAEL, HAD, STA CAASMI, HSN, SIS, EWB	All groups have a reduction in HbA1c level	Usual care
Karhula et al. (2015), Finlan d[50] To assess effectiveness of phone-based health coaching program, on improvement in HRQL and other clinical measures of T2DM and heart disease patients	250 (217); 66.3; 44.4	12	TET,ERMP/EA	Health coaches	(8), HbA1c, BP, BMI, Waist circumference, Triglycerides, Cholesterol, LDL, HDL	No statistically significant difference found in relation to HbA1c between the two groups	Usual care
Kempf et al. (2017), German y[51] To assess effectiveness of the Telemedical Lifestyle intervention Program (TeLiPro) on HbA1c	202(167/133);59.6;49	12 M(3 + 9)	TEL,ERPM/EA	Diabetes coaches	(6), HbA1c, BMI, CVD, QoL, eating behaviour, Antidiabetic medication	The difference between the two groups was statistically significant favouring the TeLiPro group in relation to HbA1c (mean ± SD - 1.1 ± 1.2%, *P* < 0.0001)	Usual Care
Odnoletkova et al. (2016), Belgiu m[52] To test the effectiveness of tele-coaching intervention on HbA1c with T2DM	574 (486); 63.1; 38.5	18 M (6+ 12)	TEL	Nurse educator	(9) HbA1c, total cholesterol, LDL cholesterol, HDL cholesterol, Triglycerides, Systolic blood pressure, Diastolic blood pressure, BMI, Weight	The difference in the means between the two groups was statistically significant favouring the coaching group.	Usual Care
Varney et al. (2014), Australi a[53] To evaluate the health coaching intervention’s long-term efficacy	94 (71); 64.1; 31.9	12 M (6 + 6)	TEL	Registered dietician	(13) HbA1C, Fasting glucose, cholesterol LDL cholesterol, HDL cholesterol, Triglyceride, Systolic BP, Diastolic BP, Weight, BMI, Waist circumference Physical activity, K10 depression score	Significant effects were observed between groups at 6 months in relation to HbA1C (reductions in A1C up to 0.8%)(*P* = 0.03)	Usual Care

*ERPM/EA* electronic remote patient monitoring/electronic assistance, *FTF* face to face, *GRP* group, *TEL* telephone, *CAL* clinical attachment loss, *TBSES* tooth-brushing self-efficacy, *FVSS* Fruit and Vegetable Screener score, *SF-12* Short-Form Health Survey, *PHQ* Patient Health Questionnaire, DDS Diabetes Distress Scale, *PAEL* Positive and active engagement in life, *HAD* Health-directed activity, *STA* Skill and technique acquisition, *ADS* Appraisal of Diabetes Scale, *HDL* High-density lipoprotein, *CAASMI* Constructive attitudes and approaches Self-monitoring and insight, *ISEL-12* Interpersonal Support Evaluation List, *HSN* Health service navigation, *SIS* Social integration and support, Emotional well-being EWB, *LDL* Low-density lipoprotein, *HTS* Healthcare team support, *RSC* Reinforcement for self-care, *ASC* Awareness of self-care goals, *ASK* Adherence Starts with Knowledge, *MAS* Morisky Adherence Scale, *PAM* Patient Activation Measure,), *BFS* Benefit-Finding Scale, *PSS-4* Perceived Stress Scale, *ADDQoL-19* 19-item Audit of Diabetes-Dependent Quality of Life scale

Effect sizes (Cohen’s ​*d*) [54] for the included interventions were calculated in line with recommended procedures for pretest-posttest-control group designs (i.e., RCTs with pre- and post- measures of the outcome variable) [55] which control for baseline differences in the outcome measure. In particular, baseline mean HbA1c values were subtracted from follow-up mean values for the intervention and control groups, separately, and these new values used to compute the effect size difference. Baseline standard deviations were used to estimate the pooled standard deviation to account for the fact that, *if* the intervention changes the outcome at follow-up, variation in outcome scores is likely to be greater in the intervention compared to control group. An Excel spreadsheet was created to calculate effect size differences following Morris’ (16) formula based on data reported in the papers. Where baseline scores were not reported, effect sizes were based on follow-up scores using software available at www.psychometrica.de. The effect sizes were calculated so that positive effect sizes indicated greater reductions in HbA1c in the intervention group compared to the control group. As per Cohen’s guidelines, the intervention has a small effect size when d ≥ 0.20, a medium effect size when d ≥ 0.50, and a large effect size when d ≥ 0.80. Effect sizes of d < 0.20 were considered to be trivial.

### Behaviour Change Technique (BCT) coding

The BCT taxonomy [25] was applied to the included studies to identify the use of BCTs. Two independent researchers (AA, HH) coded the intervention content reported in the methods section (intervention description) of each paper against the BCT taxonomy version 1 (BCTTv1), to identify the BCTs used in the health coaching interventions [25]. The coders followed the BCTTv1 guidance, for example, if a BCT was unclear (present or absent), it was coded as absent, as per the BCTTv1 guidance [25]. Both coders used Microsoft Excel (version 16.66.1) to generate a list of identified BCTs across all included interventions. Several discussion meetings were held to discuss the BCTs identified and to resolve any disagreements regarding the coded BCTs until reaching an agreement. A third independent reviewer (PN) was involved to confirm consensus decisions.

### Meta-analytic strategy

Meta-Essentials version 1.5 [56] was used to compute the sample-weighted average effect (Hedges *g*_+_) of the health coaching interventions on HbA1c scores. Cochrane’s *Q* was used to test whether the effect sizes were heterogeneous and the *I*^2^ statistic was used to assess the proportion of the variance in the effect sizes explained by any heterogeneity. Moderator analyses were then conducted to identify variables that accounted for any variability in effect sizes. For categorical moderators (e.g., presence or absence of a BCT) average effect sizes were calculated for each level of the moderator. The difference between the effect sizes was assessed using the *Q* statistic. The significance of continuous moderators was tested using meta-regression (see Tables 2 & 3).

**Table 2:** Sample-weighted Average Effect Sizes (ES) for Interventions Including vs. Excluding Specific BCTs

BCT No.	BCT	*k*	*g*_+_ present (95% CI)	*g*_+_ absent (95% CI)	Q for difference	*p*
1.1	Goal setting (behaviour)	13	0.26 (0.12, 0.41)	0.33 (0.18, 0.47)	0.38	0.538
1.2	Problem solving	10	0.19 (0.07, 0.30)	0.37 (0.21, 0.52)	3.51	0.061
1.3	Goal setting (outcome)	7	0.35 (0.25, 0.45)	0.25 (0.11, 0.40)	1.01	0.315
1.4	Action planning	8	0.25 (0.08, 0.42)	0.32 (0.18, 0.46)	0.44	0.506
1.5	Review behaviour goal(s)	3	0.20 (−0.25, −0.64)	0.31 (0.21, 0.42)	0.37	0.545
1.6	Discrepancy between current behaviour and goal	2	0.46 (−0.09, 1.00)	0.27 (0.17, 0.38)	0.42	0.519
1.7	Review outcome goal(s)	1	–	–	–	–
1.8	Behavioural contract	1	–	–	–	–
2.1	Monitoring of behavior by others without feedback	1	–	–	–	–
2.2	Feedback on behaviour	1	–	–	–	–
2.3	Self-monitoring of behaviour	3	0.22 (−0.28, 0.73)	0.29 (0.19, 0.39)	0.06	0.808
2.4	Self-monitoring of outcome(s) of behaviour	5	0.23 (−0.05, 0.52)	0.30 (0.19, 0.41)	0.18	0.672
2.5	Monitoring of outcome(s) of behavior without feedback	1	–	–	–	–
2.6	Biofeedback	5	0.18 (−0.11, 0.46)	0.32 (0.22, 0.42)	0.82	0.365
2.7	Feedback on outcome(s) of behaviour	4	0.28 (−0.03, 0.58)	0.29 (0.18, 0.40)	0.00	0.951
3.1	Social support (unspecified)	8	0.30 (0.14, 0.45)	0.28 (0.13, 0.43)	0.02	0.884
3.3	Social support (emotional)	2	0.33 (0.12, 0.55)	0.29 (0.17, 0.40)	0.11	0.746
4.1	Instruction on how to perform a behaviour	1	–	–	–	–
8.7	Graded tasks	1	–	–	–	–
9.1	Credible source	5	0.08 (−0.04, 0.19)	0.34 (0.22, 0.46)	7.67**	**0.006**
10.4	Social reward	3	0.01 (−0.20, 0.22)	0.32 (0.21, 0.43)	3.92*	**0.048**
12.5	Adding objects to the environment	1	–	–	–	–
13.2	Framing/reframing	2	0.10 (−0.35, 0.54)	0.31 (0.20, 0.42)	0.82	0.365

**Table 3:** Moderators of the Effect of Health Coaching Interventions for T2DM: Sample-weighted Average Effect Sizes (ES)

						Categorical	Continuous		
Moderators	*N*	*k*	Levels of the moderator	*Q*	*p*	*g +* (95% CI)	*β*	SE	*p*
*Sample moderators*									
Age (in years)	2928	18					0.19	0.01	0.442
Gender (percentage of females)	2857	17					−0.13	0.00	0.603
*Methodological moderators*									
Number of BCTs used	3222	20					−0.36	0.02	0.107
Study length	3222	20					0.14	0.01	0.535
Intervention length	1366	6					−0.04	0.05	0.916
Follow-up length	1366	6					−0.25	0.05	0.574
Type of control group	3222	20		0.69	0.406				
	1078	6	Active control			0.24 (0.10, 0.37)			
	2144	14	Usual care			0.32 (0.417, 0.46)			
Type of intervention provider	3222	20		1.24	0.538				
	2182	12	Healthcare professional			0.25 (0.10, 0.39)			
	568	4	Coaches			0.36 (0.06, 0.65)			
	472	4	Assistants/students			0.37 (0.25, 0.48)			
Mode of Delivery	3222	20		1.17	0.556				
	1275	6	Telephone only			0.23 (0.05, 0.42)			
	1134	8	Telephone & FtF			0.36 (0.20, 0.51)			
	813	6	Other combinations			0.25 (0.01, 0.48)			
Primary outcome measure	3222	20		4.20*	**0.040**				
	2750	16	HbA1c			0.32 (0.20, 0.45)			
	472	4	Others			0.10 (0.03, 0.17)			
Theory use in intervention development	3222	20		1.34	0.247				
	2532	14	Used			0.24 (0.16, 0.32)			
	690	6	Not used			0.43 (0.15, 0.72)			
MI theory use	3222	20		0.23	0.632				
	2108	9	Used			0.26 (0.15, 0.37)			
	1114	11	Not used			0.32 (0.14, 0.50)			

Publication bias was assessed through visual inspection of the funnel plot (i.e., lack of asymmetry in the distribution of the studies) and Egger’s regression.

### Study quality

The Cochrane collaboration tool was used to assess the quality of the included studies [57].. Each study was rated based on specific criteria related to the quality of its methods and reporting, selection, performance, detection, attrition, reporting, and other biases. The assessment of study quality was evaluated by three reviewers (AA,EG,SC). See Table 4 and Fig. 2 for further details.

**Table 4:** Risk of bias assessments based on the Cochrane collaboration tool

	Random sequence generation (Selection Bias)	Allocation concealment (Selection bias)	Blinding of participants and personnel (Performance bias)	Blinding of outcome assessment (Detection bias)	Incomplete outcome data (Attrition bias)	Selective reporting (Reporting bias)	Other sources of bias (Other bias)
Frosch et al. (2011), U S[34]	Low	Low	High	High	Low	Low	Unclear
Glasgow et al. (2006), U S[35]	Unclear	unclear	Unclear	Unclear	Low	Low	High
Kim et al., (2015), U S[36]	Unclear	Unclear	High	Low	Low	Low	Unclear
McKay et al. (2002), U S[37]	Unclear	Unclear	Unclear	Low	Low	Low	Unclear
Ruggiero et al. (2010), U S[38]	Unclear	Unclear	Unclear	High	Low	Low	High
Sacco et al. (2009), U S[39]	Low	Unclear	Unclear	Low	Low	Low	Unclear
Thom et al. (2013), U S[40]	Unclear	Low	Unclear	Low	Low	Low	Unclear
Whittemore et al. (2004), U S[41]	Unclear	unclear	Unclear	unclear	Low	Low	High
Willard-grace et al. (2015), U S[42]	Low	Low	High	Unclear	Low	Low	Unclear
Wolever et al. (2010), U S[43]	Unclear	unclear	low	low	Low	Low	High
Chen et al., (2016), Taiwa n[44]	Low	low	Unclear	low	low	low	Unclear
Lin et al., (2021), Taiwa n[45]	Low	low	low	Unclear	low	low	Unclear
Basak Cinar & Schou (2014), Turke y[46]	Unclear	unclear	High	High	low	Low	Unclear
Sherifali et al., (2020 )[47]	Low	low	Unclear	Unclear	Low	Low	Low
Cho et al. (2011), Kore a[48]	Unclear	unclear	unclear	High	Low	Low	Unclear
Holmen et al. (2014), Norwa y[49]	Low	Unclear	High	Low	Low	Low	Unclear
Karhula et al. (2015), Finland,[50]	Low	Low	Unclear	Low	Low	Low	Unclear
Kempf et al. (2017), German y[51]	Low	Low	Low	Low	Low	Low	Low
Odnoletkova et al. (2016), Belgiu m[52]	Low	Low	Unclear	Low	Low	Low	Unclear
Varney et al. (2014), Australi a[53]	Low	Low	High	High	Low	Low	High

## Results

### Search Results

**Fig. 1:**
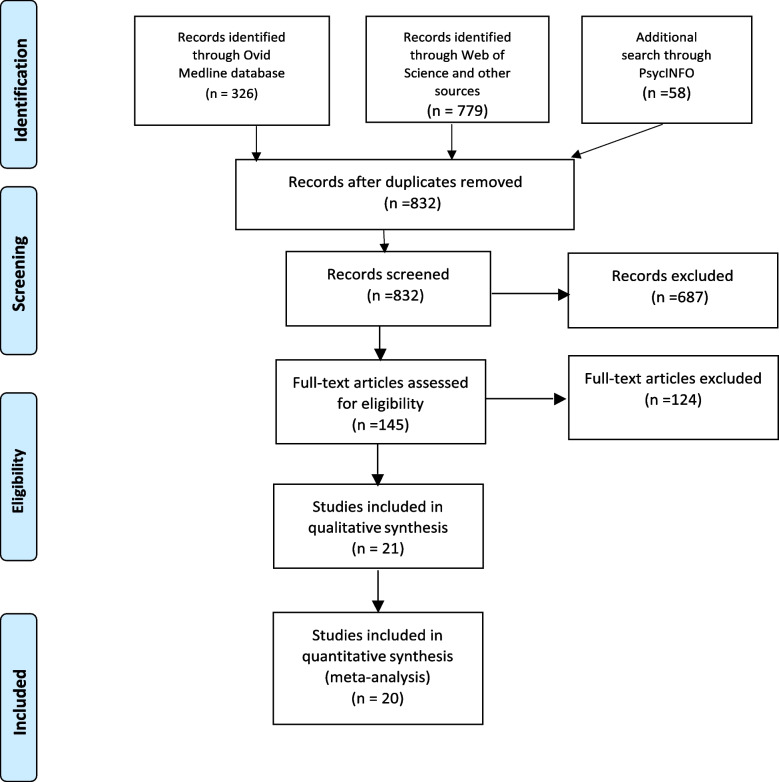
PRISMA Flow Diagram Showing Study Selection Process

The search results yielded 1163 titles and abstracts through Medline, PsycINFO and the Web of Science. There were 145 full-text studies checked for eligibility and a total of 20 RCTs met inclusion criteria (see Fig. 1) [32].

### Meta-analytic Results

Meta-analysis of 20 effect sizes from 20 unique studies, with a total sample of 3222 participants, indicated that, on average, health coaching interventions for T2DM have a small but statistically significant (positive) effect on reducing HbA1c (*g*_+_ = 0.29, 95% CI: 0.18 to 0.40). Visual inspection of the funnel plot suggested that there was no asymmetry in the distribution of the studies and no risk of publication bias. Egger’s regression was also non-significant (*p* = 0.730), indicating lack of publication bias.

The effect sizes (d) of interventions ranged from d = − 0.05 to d = 0.78. None of the interventions had a large effect size [44], and only three had a medium effect size (d = 0.71 to d = 0.78) [42, 45, 51, 53]. The remaining 17 interventions had small (d ≥ 0.20) [36, 38–40, 43, 46–49, 52] or trivial (d < 0.20) effect sizes [34, 35, 37, 41, 50]. Cochrane’s *Q* was statistically significant (*Q* = 36.68, *p* = .009) suggesting that the effect sizes were heterogeneous and the *I*^2^ statistic indicated that a proportion of the variance in the effect sizes was explained by this heterogeneity (*I*^2^ = 48.20%), which indicates a need for moderation analysis to identify variables that account for the variability.

### Study Characteristics

Table 1 reports the characteristics of included studies for both interventions (health coaching), and control groups (usual care), including sample size, mean age of participants, intervention duration, personnel, and mode of delivery (e.g., face-to-face, telephone-based, web-based). The included studies comprised 20 RCTs published between 1950 and 2022. A total of 3222 participants were included in the 20 studies, of whom 1674 were randomised to receive coaching interventions and 1548 were allocated to control groups. The majority of studies (*n* = 10) were conducted in the US [34–43], two were conducted in Taiwan [44, 45], and the rest were conducted once in different countries including Turkey [46], Canada [47], South Korea [48], Norway [49], Finland [50], Germany [51], Belgium [52], and Australia [53]. In the 17 studies that reported gender of participants, 53% of participants were female. The mean age of the recruited participants was 59.3 (SD = 6.2). Due to the inconsistent reporting of other demographic and socioeconomic characteristics, such as education, ethnicity and income status, across the 20 papers we were unable to report them here. The recruitment of participants was varied and drawn from different communities including ethnic community centres [36], community health centres [34, 48, 49], community advertisement [43, 47, 49, 51], primary care or hospital clinics [38, 41, 45, 46, 53] and databases [40, 44, 50, 52]. For clinical factors, including HbA1c, there were no discernible changes between the intervention and control groups at baseline. The mean HbA1c level across all studies at baseline was 8.42% (SD = 0.78). The reduction in HbA1c found to be clinical significant in eight studies [36, 40, 42–44, 46, 47, 51] (decrease of ≥5 mmol/mol )[58].

Moderation analysis of the sample characteristics indicated that intervention effectiveness was not related to age (*β* = 0.19, *p* = 0.442) or gender (*β* = − 0.13, *p* = 0.603). Moderation analysis of the study characteristics indicated that only the type of primary outcome measure was significantly related to intervention effectiveness (*Q* = 4.20, *p* = 0.040), such that studies including HbA1c as the primary outcome (*g*_+_ = 0.32, *k* = 16) were more effective than studies with other primary outcomes (*g*_+_ = 0.10, *k* = 4).

### Mode of delivery and intervention duration

Health coaching was delivered through various methods including exclusive telephone-based [34, 39, 43, 47, 52, 53], exclusive web or mobile-based remote patient monitoring/electronic assistance (ERPM/EA) systems [37] or in combinations of face-to-face and telephone-based [36, 38, 40, 42, 44–46]; face-to-face and ERPM/E A[48] telephone-based and ERPM/EA [49–51] or face-to-face, telephone-based and ERPM/EA [35, 41]. The duration of studies ranged from two [37] [48] to 18 months [52] (*Mdn* = 6 months). Only six studies reported separate figures for intervention and follow-up durations, with intervention duration ranging from three [51] to 10 months [46] (*Mdn* = 6 months) and the duration of follow-ups ranging from six [46] to 12 months [52] (*Mdn* = 7 months). Mode of delivery (*Q* = 1.17, *p* = 0.556) and the duration of study (*β* = 0.14, *p* = 0.535), intervention (*β* = − 0.04, *p* = 0.916) and follow-up (*β* = − 0.25, *p* = 0.574) were not significantly related to intervention effectiveness (see Table 3).

### Delivery personnel

Different people delivered the health coaching interventions. In four studies, the health coaching intervention was delivered by untrained personnel [34, 41, 44, 46, 53], while the remaining 16 interventions reported training of the interventionist on health coaching. Seven studies relied on nurses to deliver coaching sessions [34, 36, 47–49, 52], four studies provided interventions by trained health coaches [35, 37, 50, 51], and only one study was delivered by health coaches certified by the International Coach Federation (ICF) [45]. The remaining interventions were delivered by different professionals, including dental care providers [46], community health workers [36], dieticians [53], medical staff [38, 42], pharmacists [44], psychologists [43], college students [41], peer patients [40], and physicians [48]. Type of intervention provider was not significantly related to intervention effectiveness (*Q* = 1.24, *p* = 0.538) (see Table 3).

### Behavioural framework and theory use

The heterogeneity of interventions was evident in relation to the employed approaches and underpinning theories. Out of the 20 papers, five studies did not report the use of theories [34, 37, 44, 48, 51, 53]. The remaining 15 were grounded in different theories or frameworks. Most studies employed motivational interviewing [35, 36, 40, 42, 45–47, 49, 52], two studies used the transtheoretical model [38, 49], and self-efficacy theory, cognitive-behavioural therapy and social-cognitive theory were each used once [39, 46]. The use of theory was not significantly related to intervention effectiveness (*Q* = 1.34, *p* = 0.247), nor was the specific use of MI (*Q* = 0.23, *p* = 0.632) (see Table 3).

### Identified BCTs

A total of 23 BCTs were identified across the 20 studies reviewed (see Table 5). Interventions were varied in terms of the number of BCTs that were utilized in each intervention, ranging from 0 to 9 BCTs. The median of BCTs used across all interventions was 5. The most frequently coded BCT was 1.1 *goal setting (behaviour)*, which has identified in 13 interventions [34–36, 38–41, 45, 46, 49–51]. 1.2 *problem solving* was the second most commonly identified BCT, reported in 10 interventions [35–39, 41, 43, 49, 52, 53]. Two BCTs, *1.4 action plan* [34, 35, 39, 40, 45, 46, 50, 53] and *3.1 social support (unspecified)* [35, 37–39, 44, 45, 47, 48], were each reported in eight studies. *1.7 review outcome goals, 1.8 behavioural contract, 2.2 feedback on behaviour, 4.1 instruction on how to perform a behaviour, 8.7 graded tasks, 12.5 adding objects to the environment, and 2.5 monitoring outcome(s) of behaviour by others without feedback* were each used once in six interventions [37, 39, 46, 48, 52, 53]. No BCTs were identified in one study [42].

**Table 5:** Behaviour Change Techniques (BCTs) used in each intervention

	Effect size	*Underpinning Theory*	*1.1goal setting (behaviour)*	*1.2 problem-solving*	*1.3 goal- setting (outcome)*	*1.4 action planning*	*1.5 review behaviour goal(s)*	*1.6 Discrepancy between current behavior and goal*	*1.7 Review outcome goals*	*1.8 behavioural contract*	*2.1 monitoring of behaviour by others without feedback*	*2.2 feedback on behaviour*	*2.3 Self-monitoring of behaviour*
Frosch et al. (2011), U S [34]	− 0.05	NA	**Χ**			**Χ**	**Χ**						
Glasgow et al. (2006), U S [35]	0.062	MI, CCM, SCT	**Χ**	**Χ**		**Χ**	**Χ**						
Kim et al. (2015), U S [36]	0.292	MI, PPM	**Χ**	**Χ**	**Χ**								
McKay et al. (2002), U S [37]	0.14	NA		**Χ**									
Ruggiero et al. (2010), U S [38]	0.259	TTM	**Χ**	**Χ**									
Sacco et al. (2009), U S [39]	0.172	CBT	**Χ**	**Χ**		**Χ**		**Χ**	**Χ**				
Thom et al. (2013), U S [40]	0.383	MI	**Χ**			**Χ**							
Whittemore et al. (2004), U S [41]	0.098	ACI	**Χ**	**Χ**	**Χ**								
Willard- Grace et al. (2015), U S [42]	0.478	CCM, MI											
Wolever et al. (2010), U S [43]	0.253	DIM		**Χ**	**Χ**								
Chen et al. (2016), Taiwan [44]	0.78	NA											
Lin et al., (2021), Taiwa n [45]	0.462	MI	**Χ**		**Χ**	**Χ**					**Χ**		
Basak Cinar and Schou (2014), Turke y [46]	0.383	MI, SET	**Χ**		**Χ**	**Χ**							
Sherifali et al., (2021), Canad a [47]	0.31	MI			**Χ**								
Cho et al. (2011), Kore a [48]	0.328	NA										**Χ**	
Holmen et al. (2014), Norwa y [49]	−0.167	MI, TTM	**Χ**	**Χ**									**Χ**
Karhula et al. (2015), Finlan d [50]	0.087	PTM	**Χ**			**Χ**							**Χ**
Kempf et al. (2017), German y [51]	0.713	NA	**Χ**										**Χ**
Odnoletkova et al. (2016), Belgiu m [52]	0.19	CF,MI, PPM		**Χ**									
Varney et al. (2014), Australi a [53]	0.729	NA	**Χ**	**Χ**	**Χ**	**Χ**	**Χ**	**Χ**		**Χ**			
n(%)			13 (65)	10 (50)	7 (35)	8 (40)	3 (15)	2 (10)	1 (5)	1 (5)	1 (5)	1 (5)	3 (15)

	*2.4 self-monitoring of outcome(s) of behaviour*	*2.5 monitoring outcome(s) of behaviour by others without feedback*	*2.6 biofeedback*	*2.7 feedback on outcome(s) of behaviour*	*3.1 social support (unspecified)*	*3.3 social support (emotional)*	*4.1 instruction on how to perform a behaviour*	*8.7 Graded tasks*	*9.1credible sources*	*10.4 social reward*	*12.5 adding objects to the environment*	*13.2 framing/reframing*	# of Used BCTs
Frosch et al. (2011), U S [34]									**Χ**				4
Glasgow et al. (2006), U S [35]			**Χ**	**Χ**	**Χ**				**Χ**				8
Kim et al. (2015), U S [36]	**Χ**											**Χ**	5
McKay et al. (2002), U S [37]	**Χ**		**Χ**	**Χ**	**Χ**				**Χ**		**Χ**		7
Ruggiero et al. (2010), U S [38]					**Χ**								3
Sacco et al. (2009), U S [39]				**Χ**	**Χ**			**Χ**		**Χ**			9
Thom et al. (2013), U S [40]						**Χ**							3
Whittemore et al. (2004), U S [41]						**Χ**				**Χ**			5
Willard- Grace et al. (2015), U S [42]													0
Wolever et al. (2010), U S [43]									**Χ**				3
Chen et al. (2016), Taiwan [44]					**Χ**								1
Lin et al., (2021), Taiwa n [45]					**Χ**								5
Basak Cinar and Schou (2014), Turke y [46]		**Χ**											4
Sherifali et al., (2021), Canad a [47]					**Χ**								2
Cho et al. (2011), Kore a [48]					**Χ**				**Χ**				3
Holmen et al. (2014), Norwa y [49]	**Χ**		**Χ**							**Χ**		**Χ**	7
Karhula et al. (2015), Finlan d [50]	**Χ**		**Χ**										6
Kempf et al. (2017), German y [51]	**Χ**		**Χ**	**Χ**									5
Odnoletkova et al. (2016), Belgiu m [52]							**Χ**						7
Varney et al. (2014), Australi a [53]													7
n(%)	5 (25)	1 (5)	5 (25)	4)20)	8 (40)	2 (10)	1 (5)	1 (5)	5 (25)	3 (15)	1 (5)	2 (10)	

*MI* Motivational interviewing, *SET* self-efficacy theory, *CCM* chronic care model, *SCT* social cognitive theory, *TTM* transtheoretical model, *TM* Pfizer’s telecoaching model, *PPM* PRECEDE–PROCEED mode, CF Coach framework, *CBT* Cognitive behavioural therapy, *ACI* Adaptation to chronic illness*, DIM Duke integrative medicine*

### BCTs and intervention effectiveness

An overview of the use of different BCTs and effect sizes found in each study is presented in Table 5. The most effective intervention based on the effect size (d = 0.78) used only one BCT: *3.1 social support (unspecified)* [44]. Only one BCT, 1.1 *goal setting (behaviour,)* was used across all the interventions with a medium effect size, although it was also the most commonly used BCT across interventions with small or trivial effects.

There was no evidence of an association between the number of BCTs used in an intervention and its effect size (*β* = − 0.11, *p* = 0.651) (see Table 2). Of the moderation analysis with 23 different BCTs identified, only two analysis yielded significant results. Specifically, interventions that used *credible sources* of information (BCT 9.1) (Hedges’ *g*_+_ = 0.08, *k* = 5) were significantly less effective than interventions that did not use this BCT (Hedges’ *g*_+_ = 0.34, *k* = 15; *Q* = 7.67, *p* = 0.006). In addition, interventions that used *social reward* (BCT 10.4) (Hedges’ *g*_+_ = 0.01, *k* = 3) were significantly less effective than interventions that did not use this BCT (Hedges’ *g*_+_ = 0.32, *k* = 17, *Q* = 3.92; *p* = 0.048).

### Quality of the included studies

**Fig. 2:**
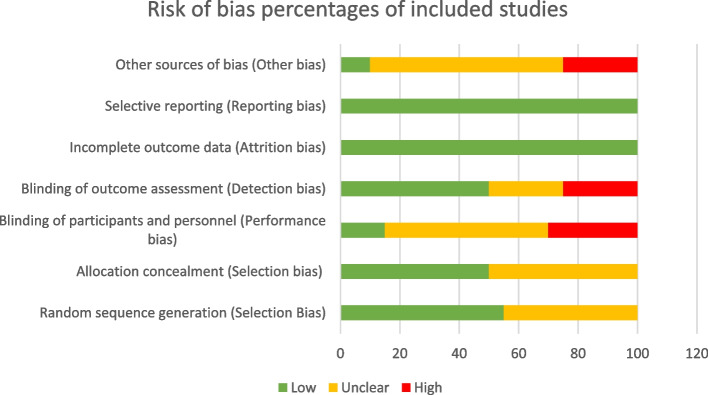
Risk of bias of included studies

Although some studies showed good methodological quality due to their low bias [44, 45, 50–52], the majority were weak because of either high or unclear risk of bias [34, 35, 37–43, 46–49, 53]. Eleven of the 20 studies [34, 39, 42, 44, 45, 47, 49–53] described the method of randomization generation and 10 studies [34, 40, 42, 44, 45, 47, 50–53] used a concealed allocation schedule. The methodological quality of blinding participants and personnel on the assignment of participants to study groups were generally low due to either high or unclear bias in procedures across most studies and insufficient detail. Across all the included studies, attrition bias and selective outcome reporting bias were low and not detected. Table 4 and Fig. 2 provide further details about the quality of the included studies.

## Discussion

This review sought to identify and investigate the use of BCTs in health coaching interventions for T2DM. The included health coaching interventions were varied in their designs, including intervention duration, session length, intervention providers, theoretical basis, BCTs utilised and delivery modality. Overall, the meta-analysis indicated that health coaching had a significant small-sized effect (*g*_+_ = 0.29) on blood glucose control. Studies that included HbA1c as the primary outcome had larger effect sizes indicating the benefit of a close correspondence between the main target of the intervention and the primary outcome.

Our meta-analysis found no advantage to utilizing one particular delivery method over others. Furthermore, no specific length of health coaching session was associated with a better outcome, although a previous,study suggested that greater time spent in coaching sessions may result in more effective result s[47]. Other studies suggest that the coaching session’s length should be framed according to the complexity of the condition presented by participants [41, 46]. Given that the conflicting pattern of findings, further research is needed to directly compare different durations of health coaching.

Interventions were delivered by different personnel, ranging from trained undergraduate students [39] to certified professional health coaches [45]. Only five out of 20 included studies relied on trained health coaches to deliver the interventions [35, 37, 45, 50, 51] while the rest were provided by people with different backgrounds including community healt h[36], dentistry [46], nutritio n[53], medicine [38, 42, 48], nursing [34, 36, 41, 48, 49, 52], pharmac y[44], psychology [43] social science [43], undergraduate student s[39], and patients’ peers [40]. This diversity may explain why coaching protocols are inconsistent or unstandardised, contributing to intervention variation and unpredictable outcomes, although the results of the meta-analysis indicated that they type of personnel delivering the health coaching did not impact on outcomes.

Theory-based interventions can lead its providers to identify the target behaviours and strategies needed to achieve desired outcomes. Half of the health coaching interventions used motivational interviewing (48%) [35, 36, 40, 42, 45–47, 49, 51, 52]. Using motivational interviewing as an intervention theoretical basis may help in understanding participants’ triggers for change and addressing their ambivalence, which is the essential goal of health coaching. Although prior studies’ findings [18, 59], suggested that employing motivational interviewing might produce better results for behaviour change, our meta-analysis findings revealed no such effect.

Considering the use of BCTs in the heath coaching interventions, we found that 19 of 20 included studies used different BCTs, with a mean of 4.5 BCTs being identified in each intervention. Although 11 of the included studies were published after the BCTTv1 was released in 2013, none explicitly reported BCTs. Out of 23 identified BCTs, only two BCTs, *goal-setting (behaviour) and problem-solving,* were commonly used across different health coaching programs with T2DM. These two BCTs have been previously identified as key ingredients for behaviour change [60], and T2DM self-management programs [29]. However, being used frequently does not imply that these BCTs contribute to improving the interventions and self-management goals [61]. For instance, the intervention with smallest effect size [34] (*d* = − 0.05) used more BCTs compared to the intervention with the largest effect size [44] (*d* = 0.78). Moreover, the meta-analysis findings failed to find any evidence linking the use of specific BCTs to greater intervention effectiveness, although most of the comparisons were based on very few studies where the BCT is present. As a result, there’s a possibility of both type 1 and type 2 errors. For example, the finding that interventions that included the BCT *social reward* had a smaller average effect size compared with studies where the BCT was absent was only based on three studies that included this BCT. In contrast, the BCT *discrepancy between current behaviour and goal,* which was found to have the largest largest effect, was not found to be a significant moderator of intervention effectiveness. However, this BCT was only identified in two studies. In sum, no clear evidence links specific BCTs to intervention effectiveness.

Overall, the heterogeneity of coaching approaches and theoretical basis utilised in the interventions, in addition to inconsistent and vague reporting of BCTs makes it challenging to identify the active intervention components. Most studies provided insufficient details about the intervention content and mechanisms, including the lack of curriculum and coaching protocol. Furthermore, none of the included studies explicitly reported the use of BCTs in interventions. Thus, it is difficult to link specific BCTs with the effectiveness or success of any included interventions. Considering that the BCT taxonomy (V1) [25] was developed in order to facilitate the systematic application and reporting of BCTs in interventions, inconsistent reporting of BCTs remains a key issue across the behaviour change and intervention development literature [62]. Consequently, interpreting and replicating some of the included interventions cannot be easily achieved due to the imprecise description of the content provided. This could be one explanation for why there is still variation in the reported effectiveness of health coaching interventions, as well as the continued replication of ineffective interventions.

Although the majority of the interventions used motivational interviewing as the underpinning theory, several BCTs that directly link to MI techniques, such as engaging techniques, focusing techniques, and evoking techniques, were completely absent as the used theoretical framework appeared to be inadequately incorporated during the interventions’ development stage [63]. These BCTs are: *verbal persuasion about capability, information about health consequences, pros and cons, comparative imagining of future outcomes, mental rehearsal of successful performance, salience of consequences, focus on past success, valued self-identity, and social comparison.*

In addition, health coaching mainly aims to enable a client to develop new personal skills, such as developing self-efficacy, self-monitoring, enhancing and valuing self-identity, self-belief, and problem-solving [17]. However, the number of potential BCTs has never or rarely been reported across interventions despite direct and strong associations with the theoretical basis of health coaching. Some examples of the relevant BCTs are *behavioural contract, commitment, monitoring of emotional consequences, anticipated regret, comparative imagining of future outcomes, identification of self as a role model, framing/reframing,* and *focus on past success*. These BCTs were rarely mentioned across many of the included studies despite their significance as core components of any health coaching intervention advocated by International Coaching Federation (2019) [64].

Finally, explicit and accurate use of BCTs and the appropriate selection of theories help to prevent frequent mistakes and incorrect replication of ineffective interventions [61]. To accurately assess an intervention’s efficacy and increase the likelihood that it will be successfully replicated, intervention developers need first to identify the intervention’s active components and whether they directly link to improvement in the outcomes. BCTs need to be explicitly specified and included in the development of new interventions as it is highly recommended to precisely guide the intervention’s procedures into effective interaction to bring about the desired behaviour change. Future studies are needed to identify the most effective BCTs to be used with health coaching interventions.

### Strengths and limitations of this review

This review has various strengths. First, it is the first review to identify the use of BCTs in health coaching studies with T2DM. Second, this review conducted a meta-analysis to investigate and evaluate the effectiveness of the BCTs in health coaching interventions and whether there is a link between using specific BCTs and reductions in Hb1Ac. Third, using the BCTs taxonomy assisted in systematically investigating and analysing interventions’ descriptions to identify the active ingredients of each intervention.

Additionally, there are several limitations to this review as well, which should be mentioned. First, it was limited to only English language papers, hence there is a possibility that some health coaching RCTs were not included. Second, studies have used various BCTs with different outcome measures, so it was difficult to determine which BCT assigned to HbA1c as an outcome. Consequently, it was difficult to be assured whether the positive results were achieved by individual BCTs or due to combinations of different BCTs. Inadequate reporting of intervention details and imprecise descriptions could lead to incorrect assumptions about the presence or absence of BCTs. Clarity and the amount of provided details on the interventions play a crucial role in coding BCTs correctly and so may have limited the accuracy of coding in the current review.

## Conclusion

This systematic review and meat-analysis examined the available evidence to determine which BCTs may be linked to improving diabetic self-management by reducing the glycaemic index. The analysis of this review showed that only 3 of the 20 interventions reported medium-sized effects on HbA1c reduction. Overall, the health coaching interventions were found to have small but significant effect on reductions in HbA1c. Whilst our findings provide some evidence to support the use of health coaching as a strategy for eliciting positive impacts on behaviours and diabetes elf-management, it may not have fulfilled its potential. Until the BCTs included in interventions are accurately reported it will be difficult to isolate the key active ingredients of health coaching interventions. Therefore, it was challenging to draw a definitive conclusion, and more research is needed to determine which BCTs are most likely to help people with T2DM control their condition. For effective and replicable health coaching interventions to be developed, the precise use and reporting of theories and BCTs is needed.

## Supplementary Information

**Additional file 1.** Supplementary Material 1 Medline search strategy.

**Additional file 2.** Supplementary Material 2 Health Coaching Studies.

## Data Availability

All data generated or analysed during this study are included in this published article.

## References

[1] Khan MAB, Hashim MJ, King JK, Govender RD, Mustafa H, Kaabi JA (2020). Epidemiology of Type 2 Diabetes – Global Burden of Disease and Forecasted Trends. J Epidemiol Glob Health.

[2] Centers for Disease Control and Prevention. Type 2 diabetes. 2021. https://www.cdc.gov/diabetes/basics/type2.html. Accessed 4 Apr 2022.

[3] Global Report on Diabetes WHO (2016). Library Cataloguing-in-Publication Data Global report on diabetes.

[4] Galaviz KI, Narayan KMV, Lobelo F, Weber MB (2018). Lifestyle and the Prevention of Type 2 Diabetes: A Status Report. Am J Lifestyle Med.

[5] Liu J, Ren Z-H, Qiang H, Wu J, Shen M, Zhang L, Lyu J (2020). Trends in the incidence of diabetes mellitus: results from the Global Burden of Disease Study 2017 and implications for diabetes mellitus prevention. BMC Public Health.

[6] Wroth SW (2015). Health Coaching Bridges Gaps in Patient Care. Alternative and Complementary Therapies.

[7] Aikaterini T, Papazafiropoulou AK, Melidonis A (2017). Type 2 diabetes and quality of life. World J Diabetes.

[8] Powell CK, Hill EG, Clancy DE (2007). The relationship between health literacy and diabetes knowledge and readiness to take health actions. Diabetes Educ..

[9] Lund SH, Aspelund T, Kirby P, Russell G, Einarsson S, Palsson O, Stefánsson E (2016). Individualised risk assessment for diabetic retinopathy and optimisation of screening intervals: A scientific approach to reducing healthcare costs. Br J Ophthalmol.

[10] Knowler WC, Barrett-Connor E, Fowler SE, Hamman RF, Lachin JM, Walker EA, Nathan DM, Watson P, Mendoza J, Smith K (2002). Reduction in the incidence of type 2 diabetes with lifestyle intervention or metformin.

[11] Lindstrom J (2006). Sustained reduction in the incidence of type 2 diabetes by lifestyle intervention: follow-up of the Finnish Diabetes Prevention Study. Lancet.

[12] Bodenheimer T, Lorig K, Holman H, Grumbach K (2002). Patient self-management of chronic disease in primary care. JAMA.

[13] Funnell MM, Anderson RM (2003). Patient empowerment: a look back, a look ahead. The Diabetes Educator.

[14] Holman H, Lorig K (2000). Patients as partners in managing chronic disease : Partnership is a prerequisite for effective and efficient health care. BMJ : Brit Med J.

[15] Wong-Rieger D, Rieger FP (2013). Health coaching in diabetes: empowering patients to self-manage. Can. J. Diabetes.

[16] Temmingh H, Claassen A, Van Zyl S, Carrara H, Dayakalashe NM, Myer L, Stein DJ (2013). The evaluation of a telephonic wellness coaching intervention for weight reduction and wellness improvement in a community-based cohort of persons with serious mental illness. J Nerv Ment Dis.

[17] Wolever RQ, Simmons LA, Sforzo GA, Dill D, Kaye M, Bechard EM, Southard ME, Kennedy M, Vosloo J, Yang N (2013). A Systematic Review of the Literature on Health and Wellness Coaching: Defining a Key Behavioral Intervention in Healthcare. Global Advances in Health and Medicine.

[18] Butterworth SW, Linden A, McClay W (2007). Health coaching as an intervention in health management programs. Disease Management & Health Outcomes.

[19] Pirbaglou M, Katz J, Motamed M, Pludwinski S, Walker K, Ritvo P (2018). Personal Health Coaching as a Type 2 Diabetes Mellitus Self-Management Strategy: A Systematic Review and Meta-Analysis of Randomized Controlled Trials. Am J Health Promot.

[20] Hill B, Richardson B, Skouteris H (2015). Do we know how to design effective health coaching interventions: A systematic review of the state of the literature. Am J Health Promot.

[21] Alamri F, Radwan N, Elolemy A, Alkhashan H (2019). Effectiveness of health coaching on diabetic patients: A Systematic Review and Meta-analysis. Tradit Med Res.

[22] Davies MJ, Heller S, Skinner TC, Campbell MJ, Carey ME, Cradock S (2008). Effectiveness of the diabetes education and self management for ongoing and newly diagnosed (DESMOND) programme for people with newly diagnosed type 2 diabetes: cluster randomised controlled trial. BMJ..

[23] van Bokhoven MA (2003). Designing a quality improvement intervention: a systematic approach. Quality and Safety in Health Care.

[24] Michie S, van Stralen MM, West R (2011). The behaviour change wheel: A new method for characterising and designing behaviour change interventions. Implement Sci.

[25] Michie S, Richardson M, Johnston M, Abraham C, Francis J, Hardeman W, Eccles MP, Cane J, Wood CE (2013). The Behavior Change Technique Taxonomy (v1) of 93 Hierarchically Clustered Techniques: Building an International Consensus for the Reporting of Behavior Change Interventions. Change Interventions Oral Medicine.

[26] Avery L, Flynn D, Van Wersch A, Sniehotta FF, Trenell MI (2012). Changing physical activity behavior in type 2 diabetes: a systematic review and meta-analysis of behavioral interventions. Diabetes Care..

[27] Cradock KA, ÓLaighin G, Finucane FM, Gainforth HL, Quinlan LR, Ginis KAM (2017). Behaviour change techniques targeting both diet and physical activity in type 2 diabetes: A systematic review and meta-analysis. Int. J. Behav. Nutr. Phys. Act.

[28] Hankonen N, Sutton S, Prevost AT, Simmons RK, Griffin SJ, Kinmonth AL, Hardeman W (2015). Which behavior change techniques are associated with changes in physical activity, diet and body mass index in people with recently diagnosed diabetes?. Ann Behav Med.

[29] Van Vugt M, De Wit M, Cleijne WHJJ, Snoek FJ. Use of behavioral change techniques in web-based self-management programs for type 2 diabetes patients: systematic review. J Med Internet Res. 2013;15(12):e279. 10.2196/jmir.2800 PMC3869055 24334230

[30] Presseau J, Ivers NM, Newham JJ, Knittle K, Danko KJ, Grimshaw JM (2015). Using a behaviour change techniques taxonomy to identify active ingredients within trials of implementation interventions for diabetes care. Implement Sci.

[31] Upsher R, Onabajo D, Stahl D, Ismail K, Winkley K (2021). The Effectiveness of Behavior Change Techniques Underpinning Psychological Interventions to Improve Glycemic Levels for Adults With Type 2 Diabetes: A Meta-Analysis. Frontiers in Clinical Diabetes and Healthcare.

[32] Liberati A, Altman DG, Tetzlaff J, Mulrow C, Gøtzsche PC, Ioannidis JP, Clarke M, Devereaux PJ, Kleijnen J, Moher D (2009). The PRISMA statement for reporting systematic reviews and meta-analyses of studies that evaluate healthcare interventions: explanation and elaboration. Br Med J.

[33] Bothwell LE, Greene JA, Podolsky SH, Jones DS, Malina D (2016). Assessing the gold standard - Lessons from the history of RCTs. N Engl J Med.

[34] Frosch DL (2011). Evaluation of a behavior support intervention for patients with poorly controlled diabetes. Arch Intern Med..

[35] Glasgow RE, Nutting PA, Toobert DJ, King DK, Strycker LA, Jex M (2006). Effects of a brief computer-assisted diabetes self-management intervention on dietary, biological and quality-of-life outcomes. Chronic Illn.

[36] Kim MT, Kim KB, Huh B, Nguyen T, Han H-R, Bone LR (2015). The effect of a community-based self-help intervention. Am J Prev Med..

[37] McKay HG, Glasgow RE, Feil EG, Boles SM, Barrera M (2002). Internet-based diabetes self-management and support: initial outcomes from the diabetes network project. Rehabil Psychol..

[38] Ruggiero L, Moadsiri A, Butler P, Oros SM, Berbaum ML, Whitman S (2010). Supporting diabetes self-care in underserved populations. Diabetes Educ..

[39] Sacco WP, Malone JI, Morrison AD, Friedman A, Wells K (2009). Effect of a brief, regular telephone intervention by paraprofessionals for type 2 diabetes. J Behav Med..

[40] Thom DH, Ghorob A, Hessler D, De Vore D, Chen E, Bodenheimer TA (2013). Impact of peer health coaching on glycemic control in low-income patients with diabetes: a randomized controlled trial. Ann Fam Med..

[41] Whittemore R, Melkus GDE, Sullivan A, Grey M (2004). A nurse-coaching intervention for women with type 2 diabetes. Diabetes Educ..

[42] Willard-Grace R, Chen EH, Hessler D, DeVore D, Prado C, Bodenheimer T (2015). Health coaching by medical assistants to improve control of diabetes, hypertension, and hyperlipidemia in low-income patients: a randomized controlled trial. Ann Fam Med..

[43] Wolever RQ, Dreusicke M, Fikkan J, Hawkins TV, Yeung S, Wakefield J (2010). Integrative health coaching for patients with type 2 diabetes. Diabetes Educ..

[44] Chen J-H, Ou H-T, Lin T-C, Lai EC-C, Yang Kao Y-H (2016). Pharmaceutical care of elderly patients with poorly controlled type 2 diabetes mellitus: a randomized controlled trial. Int J Clin Pharm.

[45] Lin CL, Huang LC, Chang YT, Chen RY, Yang SH (2021). Effectiveness of health coaching in diabetes control and lifestyle improvement: a randomized-controlled trial. Nutrients..

[46] Basak Cinar A, Schou L (2014). Health promotion for patients with diabetes: health coaching or formal health education?. Int Dent J..

[47] Sherifali D, Brozic A, Agema P, Punthakee Z, McInnes N, O'Reilly D, Usman Ali RM, Ibrahim S, Gerstein HC (2021). Effect of Diabetes Health Coaching on Glycemic Control and Quality of Life in Adults Living With Type 2 Diabetes: A Community-Based, Randomized. Can J Diabetes.

[48] Cho J-H, Kwon H-S, Kim H-S, Oh J-A, Yoon K-H (2011). Effects on diabetes management of a health-care provider mediated, remote coaching system via a PDA-type glucometer and the internet. J Telemed Telecare..

[49] Holmen H, Torbjørnsen A, Wahl AK, Jenum AK, Småstuen MC (2014). A Mobile Health Intervention for Self-Management and Lifestyle Change for Persons With Type. JMIR mHealth and uHealth.

[50] Karhula T, Vuorinen A-L, Rääpysjärvi K, Pakanen M, Itkonen P, Tepponen M, et al. Telemonitoring and mobile phone-based health coaching among Finnish diabetic and heart disease patients: randomized controlled trial. J Med Internet Res. 2015;17(6):e153. 10.2196/jmir.4059 PMC4526947 26084979

[51] Kempf K, Altpeter B, Berger J, Reuß O, Fuchs M, Schneider M (2017). Efficacy of the telemedical lifestyle intervention program TeLiPro in advanced stages of type 2 diabetes: a randomized controlled trial. Diabetes Care..

[52] Odnoletkova I, Goderis G, Nobels F, Fieuws S, Aertgeerts B, Annemans L (2016). Optimizing diabetes control in people with Type 2 diabetes through nurse-led telecoaching. Diabet Med..

[53] Varney JE, Weiland TJ, Inder WJ, Jelinek GA. Effect of hospital-based telephone coaching on glycaemic control and adherence to management guidelines in type 2 diabetes, a randomised controlled trial. Intern Med J. 2014;44(9):12515. 10.1111/imj.12515 24963611

[54] Cohen J (1988). Statistical power analysis for the behavioral sciences.

[55] Morris SB (2008). Estimating effect sizes from pretest-posttest-control group designs. Organ Res Methods.

[56] Suurmond R, van Rhee H, Hak T (2017). Introduction, comparison, and validation of Meta-Essentials: a free and simple tool for meta-analysis. Res Synth Methods.

[57] Higgins JPT, Altman DG, Gotzsche PC, Juni P, Moher D, Oxman AD, et al. The Cochrane Collaboration's tool for assessing risk of bias in randomised trials. BMJ. 2011;343(2). 10.1136/bmj.d5928 PMC3196245 22008217

[58] Lameijer A, Fokkert M, Edens M, Slingerland R, Bilo H, van Dijk P (2020). Determinants of HbA1c reduction with FreeStyle Libre flash glucose monitoring (FLARE-NL 5). J. Clin. Transl. Endocrinol..

[59] Olsen JM, Nesbitt BJ (2010). Health Coaching to Improve Healthy Lifestyle Behaviors: An Integrative Review. Am J Health Promot.

[60] Michie S, Abraham C, Whittington C, McAteer J, Gupta S (2009). Effective techniques in healthy eating and physical activity interventions: a meta-regression. Health Psychol.

[61] Prestwich A, Sniehotta FF, Whittington C, Dombrowski SU, Rogers L, Michie S (2014). Does theory influence the effectiveness of health behavior interventions? Meta-analysis. Health Psychol.

[62] Michie S, Johnston M (2012). Theories and techniques of behaviour change: Developing a cumulative science of behaviour change. Health Psychol Rev.

[63] Hardcastle SJ, Fortier M, Blake N, Hagger MS (2017). Identifying content-based and relational techniques to change behaviour in motivational interviewing. Health Psychol Rev.

[64] International Coaching Federation. ICF core competencies. 2022. https://coachingfederation.org/credentialsand-standards/core-competencies. Accessed 10 Apr 2022.

