# Supplementary figures and images for: Behavioural Change Techniques in Health Coaching-Based Interventions for Type 2 Diabetes: A Systematic Review and Meta-Analysis
Source: BMC Public Health. 2023 Jan 13;23:95. doi: 10.1186/s12889-022-14874-3 (PMC9837922; doi:10.1186/s12889-022-14874-3)

Supplementary Material 1: Medline search strategy:


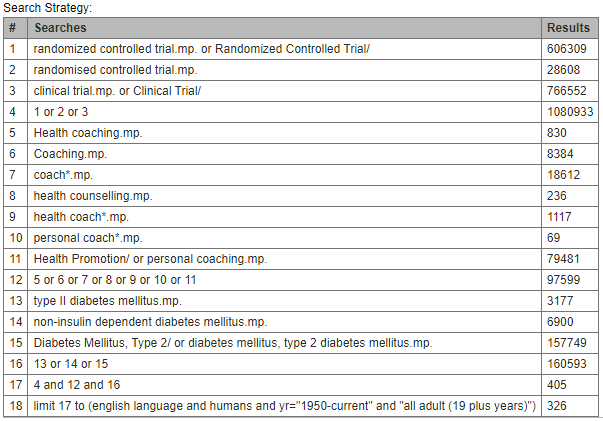

Supplement: Supplementary file 1 — Additional file 1. Supplementary Material 1 Medline search strategy. [file 12889_2022_14874_MOESM1_ESM.docx]
